# Can magnetic resonance imaging replace conventional computerized tomography for follow-up of patients with testicular cancer? A systematic review

**DOI:** 10.1007/s00345-022-03931-6

**Published:** 2022-01-17

**Authors:** Jonas Busch, Stefanie Schmidt, Peter Albers, Julia Heinzelbecker, Sabine Kliesch, Julia Lackner, David Pfister, Christian Ruf, Christian Winter, Friedemann Zengerling, Dirk Beyersdorff

**Affiliations:** 1 Department of Urology, Charité Universitaetsmedizin Berlin, Berlin, Germany; 2 Department of Urology, Vivantes Klinikum Am Urban, Dieffenbachstr. 1, 10967 Berlin, Germany; 3 UroEvidence@Deutsche Gesellschaft Für Urologie, Berlin, Germany; 4 Department of Urology, University Hospital Düsseldorf, Düsseldorf, Germany; 5 Department of Urology and Paediatric Urology, Saarland University Medical Centre and Saarland University Faculty of Medicine, Homburg, Saar Germany; 6 Department of Clinical and Surgical Andrology, Centre of Reproductive Medicine and Andrology, University Hospital, Münster, Münster, Germany; 7 Department of Urology, University Hospital Cologne, Cologne, Germany; 8 Department of Urology, Bundeswehrkrankenhaus (German Federal Armed Forces Hospital), Koblenz, Germany; 9 Urologie Neandertal (Regional Joint Practice), Erkrath, Germany; 10 Department of Urology, University Hospital Ulm, Ulm, Germany; 11 Clinic and Polyclinic for Diagnostic and Interventional Radiology and Nuclear Medicine, University Hospital Hamburg-Eppendorf, Hamburg, Germany

**Keywords:** Testicular cancer, Follow-up care, Computerized tomography imaging, Magnetic resonance imaging, Recurrence, Metastasis

## Abstract

**Purpose:**

Follow-up protocols for patients with testicular cancer (TC) have significantly reduced the number of cross-sectional imaging studies to reduce radiation exposure. At present, it is unclear whether magnetic resonance imaging (MRI) could replace conventional computerized tomography (CT) imaging. The objective of this study is to summarize the scientific evidence on this topic and to review guideline recommendations with regard to the use of MRI.

**Methods:**

A systematic literature review was performed searching Medline and Cochrane databases for prospective studies on patients with TC in the follow-up care (last search in February 2021). Additionally, guideline recommendations for TC were screened. Data extraction and quality assessment of included studies were performed and used for a descriptive presentation of results.

**Results:**

A total of four studies including two ongoing trials were identified. Overall, the scientific evidence of prospective comparative studies is based on 102 patients. Data suggest that abdominal imaging with MRI can replace conventional CT for detection of lymph node metastasis of the retroperitoneum to spare radiation exposure and contrast media application. However, experienced radiologists are needed. Clinical guidelines are aware of the risk of diagnosis-induced secondary malignancy due to CT imaging and some have adapted their recommendations accordingly. Results of the two ongoing trials on 738 patients are expected soon to provide more reliable results on this topic.

**Conclusions:**

There is growing evidence that abdominopelvic MRI imaging can replace CT imaging during follow-up of patients with TC in order to reduce radiation exposure and diagnosis-induced secondary malignancy.

## Introduction

Currently, there is no standardized international consensus on follow-up schemes for patients with testicular cancer (TC) in full remission after curative therapy. The aim of regular follow-up examinations is the detection of recurrences, as well as the early detection of late effects (harms) from previous treatment(s). But to date, there are significant differences in the recommendations from international clinical practice guidelines concerning the frequency and type of imaging procedures [1–3].

Usually, follow-up examinations after curative therapy include a clinical examination including the determination of body mass index and blood pressure and a sonography examination of the remaining testis, especially if no contralateral testicle biopsy has been performed in younger patients (< 30 years) and in patients with small testicle volume (< 12 ml) [4–6]. Sonography of the residual testicular parenchyma is also mandatory in organ-preserving procedures after tumor resection.

In addition, the control of serum tumor markers, chest X-ray imaging and abdominal and pelvic sectional imaging are generally recommended [4–6]. However, the routine use of a thoracic X-ray examination is currently questioned, especially in stage I disease, due to the late effects of radiation exposure [7]. Additionally, an expanded blood count with determination of testosterone, luteinizing hormone (LH) and lipid values should be performed once a year [8]. Further examinations should be discussed with the patient and depend on individual conditions and prior treatments.

Traditionally, computerized tomography (CT) scans (especially contrast-enhanced CTs CECT as the most sensitive) of the abdomen and pelvis are the imaging procedures of choice for the detection of retroperitoneal lymph node metastasis or recurrence, mainly because of high reproducibility and excellent imaging of the para-aortic and para-caval regions. Difficulties in diagnosis with CT scans might arise in men with little retroperitoneal fat, which tend to be an impediment for the correct interpretation of results, and might also provide false-negative results in up to 30% of cases, due to difficulties in the interpretation of lymph nodes based on morphology and size alone [9, 10]. The greatest concern, however, is the radiation exposure in patients with testicular cancer undergoing several repeated cross-sectional imaging at a young age, which is associated with a significant risk of secondary malignancies in the upcoming years [11]. The reference values (CTDI Vol) for an examination of the upper abdomen and lower abdomen with pelvis are each given as 15 mGy. A complete abdominal imaging from the upper abdomen to the pelvis, thus, typically reaches an effective dose of 10 mSv.

Therefore, magnetic resonance imaging (MRI) might be an alternative to CT scans. MRI scans were usually restricted to patients with contraindications to CT or to whom intravenous contrast media cannot be given [9, 10, 12], as they do not provide additional clinical information over CT scans. However, improved MRI technique, e. g. diffusion-weighted imaging, is a MRI technique that improves the identification of lymph nodes on the basis of degree of restricted diffusion, but it is still limited by significant overlap between benign and malignant lymph nodes [13]. Further general disadvantages of MRI are longer examination time, higher costs, and lower availability in the medical setting [14]. Side effects such as systemic nephrogenic fibrosis and intracerebral gadolinium (Gd) deposition are only to be expected with severely impaired glomerular filtration rate but appear extremely rare due to the switch to macrocyclic Gd contrast media.

In 2009, a literature review of Hansen et al. did not reveal valid data regarding the diagnostic accuracy of MRI imaging compared with current multislice CT for the diagnosis of retroperitoneal spread in testicular cancer [15]. However, the latest updates of several clinical practice guidelines include modifications concerning the frequency of a CT scan in favor of an MRI examination, as replacing CT by MRI scan would reduce the overall radiation exposure and, therefore, the risk of a radiation-induced second cancer, especially when using repeated cross-sectional imaging in men at a young age.

The purpose of this study was to update the evidence and review whether MRI could replace conventional CT imaging in patients with TC after curative therapy.

## Methods

This work was based on a systematic literature search that was conducted for the elaboration of the first German clinical practice guideline [3, 16]. In this context, several systematic literature searches were conducted. Here, we present the results combined with an updated search.

### Systematic literature search

We performed a systematic literature review to identify studies comparing the use of MRI versus CT scan in the follow-up care of men (≥ 18 years) with a proven diagnosis of TC after primary therapy. We used the biomedical databases Medline (via Ovid) and Cochrane Central Register of Controlled Trials (search period January 2010 to February 2021). Our search was limited to full text publications and to those written in English and German language. We considered clinical trials and prospective observational studies. Case control studies, case reports, case series, editorials, comments and conference abstracts were excluded. An additional search for unpublished data and ongoing studies was conducted in clinical trial registers (clinicaltrial.gov/ and www.who.int/ictrp/). We contacted the study coordinators in case of missing information for studies identified in the trial registries and hand-searched the reference lists of included studies to determine additional, potentially relevant studies.

We also searched for clinical practice guidelines in order to analyze how current evidence is translated into clinical recommendations. Therefore, we consulted well-known institutions, which provided clinical practice guidelines in the field of urology and downloaded these.

### Literature screening, data extraction and quality assessment

One review author screened the titles and abstracts and afterwards the full texts of the retrieved references and determined the relevance for inclusion. For included studies, one author extracted relevant data in evidence tables. The study quality was determined using the QUADAS-2 tool for the assessment of diagnostic accuracy [17] and the level of evidence was rated according to the Oxford criteria [18]. Clinical practice guidelines were rated with the AGREE II tool [19]. Only guidelines were reported showing a minimum quality standard of at least 50 points. In any case of uncertainty another review author was involved in each of the above-mentioned steps and a consensus was reached by discussion.

## Results

### Evidence from primary studies

**Fig. 1:**
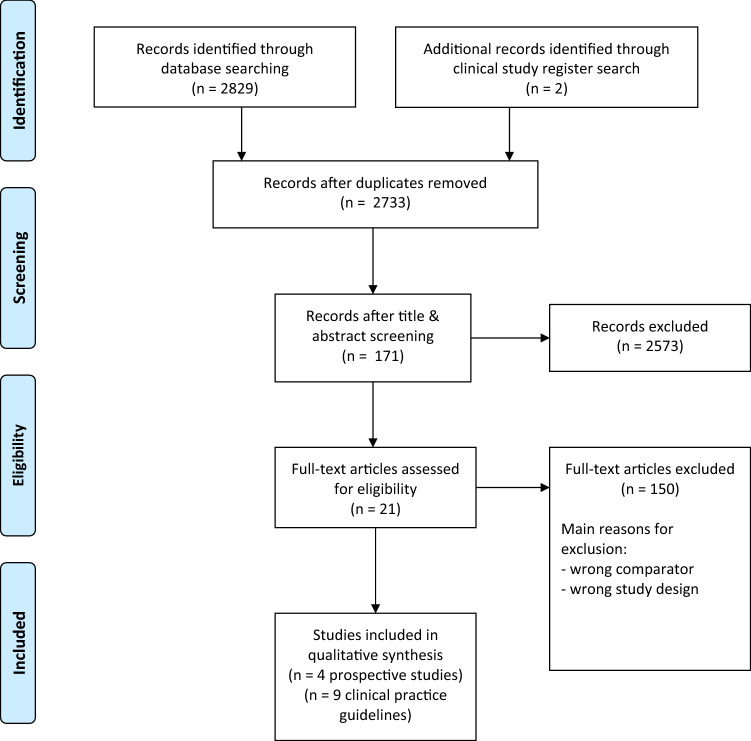
Study flow diagram of identified references

Our systematic literature review identified four prospective studies, which compared MRI to CT (Laukka et al. [20], Sohaib et al. [21], TRISST trial [22], TENY trial [23]) (Table 1; Fig. 1). The Sohaib study was added because of its high relevance although it was initially not included in the search period.

**Table 1:** Study characteristics

References	Design Number of patients Country Follow-up	Patients	Intervention	Control	Results	Level of evidence Risk of bias Additional comments
Laukka 2020	Prospective study *n* = 50 Finland Median follow-up 52 mo (17–72 mo)	Testicular cancer patients Mean age: 33 y (20–65 y) *n* = 30 (60%) stage 1 *n* = 46 patients with retroperitoneal metastases *n* = 4 patients without retroperitoneal metastases	MRI	CT	Number of metastatic lymph nodes found (mean, median, range) MRI: 1, 2, 0–9 CT: 1, 2.1, 0–10 Smallest lymph node (mm) (Mean, median, range) MRI: 12, 14.3, 7–30 CT: 12, 14.7, 8–28 No statistically significant difference in The sizes of lymph nodes (* p* = 0.277) Sensitivity of MRI 98% (95% CI 88.5–99.9%) One control patient was falsely positive in CT, but correctly classified as negative on MRI False negative MRI: 1 patient CT: 2 patients	2b At risk of bias *No consecutive or random sample* No conflict of interest declared
Sohaib 2009	Prospective study *n* = 52 United Kingdom 6 weeks follow-up	Testicular cancer patients Mean age: 34 y (18–54) *n* = 22 (42%) stage 1 *n* = 30 (51 retroperitoneal nodes)	MRI	CT	Experienced radiologists (*n* = 2): Sensitivity of MRI: 97% (95% CI 80–100%) Trainee radiologist with 1-year experience (* n* = 1): Sensitivity of MRI: 80% (95% CI 61–92%)	2b At risk of bias *No consecutive or random sample* No information about conflict of interest or funding
TRISST trial (NCT00589537)	RCT recruiting until July 2014 *n* = 660 United Kingdom Planned follow-up 5 years	Patients with seminoma CS I 16 Years and older	Arm III: MRI abdomen/retroperitoneum* at 6, 12, 18, 24, 36, 48, and 60 months Arm IV: MRI abdomen/retroperitoneum* at 6, 18, and 36 months	Arm I: CT abdomen/retroperitoneum* at 6, 12, 18, 24, 36, 48, and 60 months Arm II: CT abdomen/retroperitoneum* at 6, 18, and 36 months	*No results available at the moment*	Trial Status: completed Results are expected in 2021 *Patients with a history of ipsilateral inguino-scrotal surgery also undergo imaging of the pelvis
TENY trial (NCT03436901)	Prospective open-label study *n* = 78 Denmark follow-up 1 month	testicular cancer CS II–III Age < 18 years	MRI with Diffusion-Weighted Imaging	CT	*No results available at the moment*	Trial Status: recruiting Estimated Study Completion Date: August 2021

*CT* Computed tomography, *MRI* magnetic resonance imaging, *CS* clinical stage

Laukka et al. conducted an intraindividual comparative study of MRI and CT examinations in 50 patients with TC [20]. They obtained comparable results with both methods, CT and MRI. In this study, MRI was also performed with diffusion imaging but without contrast agent application. All 46 patients with retroperitoneal metastases could be detected with both techniques. The differences related to individual lymph nodes were not statistically significant [20].

Sohaib et al. [21] conducted a prospective diagnostic study in the UK with a 6-week follow-up (level 2b evidence). 52 patients were included with a mean age of 34 years. 22 patients initially presented with stage I disease, 30 patients with retroperitoneal nodes. The authors certify a sensitivity of 97% for the detection of retroperitoneal lymph nodes for MRI imaging [95% confidence interval (CI) of 80–100%]. However, the results of the sensitivity analysis differed by the grade of experience of the performing radiologist. A trainee radiologist with 1-year experience yielded a sensitivity of 80% (95% CI of 61–92%).

The TRISST trial (NCT00589537) is a randomized trial with a 5-year follow-up and the aim of assessing whether a reduced CT schedule or MRI could be used as safe and effective alternatives to standard CT-based surveillance. The investigators plan to recruit 660 patients with stage I seminoma disease. The design of the trial includes four interventions [24]. Two arms will each use CT scan of the abdomen/retroperitoneum and 2 arms will use MRI scan of the abdomen/retroperitoneum. The two arms of the same intervention differ in terms of the follow-up protocol (6, 12, 18, 24, 36 months versus 6, 12, 18, 24, 36, 48, and 60 months). Recruitment was finished in 2014. Formal publication is planned for August 2021.

The TENY trial (NCT03436901), is a diagnostic open-label study conducted in Denmark. The study authors aim to include 78 TC patients with stage II–III disease. They try to replace CT as a follow-up imaging method with non-ionizing whole-body MRI including diffusion-weighted imaging. The study is currently recruiting patients. Results are expected earliest in August 2021.

### Recommendations from consensus conferences and clinical practice guidelines in the change of time

The literature review of clinical practice guidelines showed 9 results (Table 2). After applying the minimum quality criteria, 6 guidelines remained and are discussed in the following. We also provide information on our German guideline.

**Table 2:** Quality assessment of included clinical practice guidelines

Clinical practice guideline	Quality assessment
European Association of Urology (EAU) 2020	65/100
National Cancer Care Network (NCCN) 2020	54/100
European Society of Medical Oncology (ESMO) 2019	53/100
American College of Radiology (ACR) 2016	30/100
Alberta Health Services (AHS) 2016	48/100
Cancer Care Ontario (CCO) 2014	65/100
European Society of Medical Oncology (ESMO) 2013	34/100
Scottish Intercollegiate Guidelines Network (SIGN) 2011	82/100
Belgian Health Care Knowledge Center (KCE) 2010	71/100

Between 2010 and 2014, the Belgian Health Care Knowledge Center (KCE), the Scottish Intercollegiate Guidelines Network (SIGN) and the Cancer Care Ontario (CCO) clinical practice guidelines recommended CT scans as the primary diagnostic imaging modality for TC [25, 26]. These recommendations were accompanied by precautions that should be taken to avoid iodine allergy or nephrotoxicity as an adverse event in patients. For these patients, MRI could be an alternative follow-up technique.

In 2018, an European Society of Medical Oncology (ESMO) consensus conference on testicular cancer re-addressed the role of MRI versus CT in TC patients. Three recommendations were made for the use of MRI during staging and for post-treatment assessment of TC [27]:

- MRI may be helpful for characterization of equivocal CT findings (e.g., in liver, bone, brain). (Level of evidence: IV; Strength of recommendation: A; Level of consensus: No vote obtained)
- MRI is not routinely recommended in all patients for staging of the retroperitoneum. (Level of evidence: III; Strength of recommendation: B; Level of consensus: 94.1% (32) yes, 5.9% (2) abstain (34 voters))
- An MRI can be recommended for follow-up of the retroperitoneum, if standard protocols are used and the results are reported by an experienced radiologist. [Level of evidence: III; Strength of recommendation: A; Level of consensus: 85.3% (29) yes, 2.9% (1) no, 11.8% (4) abstain (34 voters)].

The European Association of Urology (EAU) clinical guideline from 2020 recommended contrast-enhanced CT as the most sensitive means to evaluate the thorax, abdomen and pelvis for initial TC staging. Contrast enhanced CT (CECT) was recommended in all patients for staging before orchidectomy but may be postponed until histopathological confirmation of malignancy [2]. For abdominal staging purposes, similar accuracy was shown for CECT in the detection of retroperitoneal nodal enlargement [21, 28]. The EAU stated that MRI is subject to greater artefacts and is not routinely indicated. If CT is contraindicated because of allergy to iodine-based contrast media, non-contrast CT may be performed to evaluate nodal size. Currently, there are no indications for routine use of MRI for TC staging. MRI should be used to screen for brain metastases [2, 29, 30].

The German clinical guideline on TC provides separate follow-up schemes for the different stages and types of TC [3]. In view of the lack of evidence, the guideline panel published a recommendation based on interdisciplinary expert consensus: MRI of the abdomen/pelvis should replace the CT of the abdomen/pelvis in the follow-up care of patients with TC if performed at centers with proven experience to reduce radiation exposure. Experienced radiologists are required for the interpretation of results.

The 2020 National Cancer Care Network (NCCN) guideline on TC recommends abdominal/pelvic CT scan for follow-up examinations [1]. In select circumstances, an MRI can be considered to replace an abdominal/pelvic CT scan. The MRI protocol should include visualization of the retroperitoneal and pelvic nodes and should be performed in experienced centers in interpreting MRI results for testicular cancer. The same imaging modality (CT or MRI) should be used throughout follow-up.

## Discussion

Within this systematic review, the evidence based on prospective studies regarding the question whether MRI could replace conventional CT imaging in patients with TC after curative therapy is summarized. Two prognostic studies with data on 102 patients showed that the interpretation of MRI scans by experienced radiologists showed a good sensitivity for the detection of retroperitoneal lymph nodes. Results of two ongoing clinical trials (including further patients 738) are expected urgently and might change follow-up schemes of patients with TC in the future.

Several additional studies, which did not match our inclusion criteria, have demonstrated the value of diffusion imaging for the detection of pathologically altered clinical stage (CS) in the setting of tumor disease and specifically germ cell tumors [31, 32]. In a retrospective single center analysis, Larsen et al. demonstrated in 759 consecutive patients that MRI follow-up is routinely possible for patients with TC CS I. The only exception was one patient with claustrophobia. The examination had to be terminated incompletely. Examination time was under 30 min in this setting with coronal T1-weighted images, axial T2-weighted and diffusion-weighted images. Contrast administration was omitted. For the detection of recurrence, a specificity of 97.4% and a sensitivity of 93.8% could be achieved for the retroperitoneum and pelvis [31]. The sensitivity for detecting relapse ≥ 10 mm in short axis lymph node diameter was 100%. The negative predictive value was 99.7%, the positive predictive value was 59.9% and the accuracy was 97.3%. The authors concluded that MRI of the retroperitoneum and pelvis constitutes a safe alternative to CT in the follow-up of patients with TC CS I with both a high sensitivity and a high specificity. They presented a robust MRI protocol with diffusion-weighted imaging and estimate that MRI follow-up of TC CS I can be easily implemented in most modern radiology departments [31].

Mosavi et al. were able to show that whole-body MRI with diffusion imaging is feasible in 71 patients with TC. However, the examination time was 45 min. In two patients, the examination had to be terminated due to claustrophobia. In this study, additional information could be obtained from diffusion-weighted imaging in two cases. Thus, in one case a residual retroperitoneal lymph node could be identified as such without activity, which would otherwise only be possible with a positron emission tomography–CT (PET–CT). In another case, a small lymph node, in the size standard range, which was conspicuous by its diffusion restriction, could be confirmed as a metastasis in the PET-CT [32].

Recently, a number of papers have been published on follow-up examinations that are risk-adapted to the respective situation with regard to frequency of CT examinations [6, 33, 34]. Depending on the risk assessment, recurrences should be identified at an early stage within the framework of an ideal follow-up while at the same time protecting against unnecessary radiation exposure.

### Further evidence on testicular cancer follow-up care

In a survey among German urologists on the adherence to grade “A” recommendations from the 2015 EAU guideline on TC [35], Nestler et al. [36] demonstrated that MRI of the abdomen was used more frequently in younger patients (43.9%; *n* = 164) and in cases of allergy to contrast media or renal insufficiency (46.0%; *n* = 172). Only 14.2% of urologists (*n* = 53) always used MRI for abdominal imaging, and MRI was not sufficiently available to 3.5% (*n* = 13). Similar to the initial imaging modality, MRI for follow‐up care was more often used by office urologists than by hospital‐based urologists (19.0% vs. 5.4%, *p* = 0.014) and by more experienced urologists (> 5 years) (17.2% vs. 5.9%, *p* = 0.041). The authors concluded that MRI is widely available in Germany and a valid option for radiation‐free follow‐up imaging of the abdomen if performed by radiologists experienced in oncological and abdominal MRI reading [36].

### Imaging guidelines on other abdominopelvic tumor entities

The need for lymph node metastasis detection is also key in other tumor entities. Clinical guideline recommendations may vary among those. The lack of studies comparing MRI and CT imaging is a general trait, although MRI is used in most cancer entities. For bladder cancer staging, Crozier et al. compared the different imaging modalities MRI/PET with conventional CT in a systematic review and meta‐analysis [37]. The pooled MRI sensitivity was 0.60 (95% CI 0.44–0.74) and the pooled specificity was 0.91 (95% CI 0.82–0.96), which is superior compared to CT for detection of positive lymph nodes in bladder cancer prior to cystectomy.

Lohman et al. systematically reviewed the diagnostic accuracy of CT and MRI for the detection of lymph node metastases in gallbladder cancer [38]. Due to a lack of data, no subgroup analysis comparing the diagnostic accuracy of CT vs. MRI could be performed. Therefore, the value of current imaging strategies for the pre-operative assessment of nodal status in gallbladder cancer remains unclear, especially regarding the detection of small lymph node metastasis.

A Cochrane review on melanoma reported that comparative data with CT or MRI are lacking [39]. The increasing availability of adjuvant therapies for people with melanoma at high risk of disease spread at presentation will have a considerable impact on imaging services, yet evidence for the relative diagnostic accuracy of available tests is limited.

For rectal cancer, Gao et al. compared the value of four imaging modalities in diagnosing lymph node involvement [40]. The results of the overview indicated that endoscopic ultrasound had better diagnostic value than CT and endorectal ultrasound in the diagnosis of lymph node invasion. Compared with CT and endorectal ultrasound, MRI was more sensitive. Endorectal ultrasound was more specific when compared to CT. Endoscopic ultrasound and MRI had comparable diagnostic accuracy for evaluating lymph node involvement, but no modality was particularly accurate. However, based on current technology and conditions, endoscopic ultrasound and MRI may be choices for diagnosing lymph node involvement in patients with rectal cancer.

In 2017, Liu et al. published a meta-analysis comparing CT, PET–CT, MRI and diffusion-weighted imaging MRI for detection of lymph node metastases in patients with cervical cancer [41]. The authors concluded that among the four non-invasive modalities, the PET or PET/CT had the highest specificity, and the diffusion-weighted imaging MRI had the highest sensitivity.

## Conclusions

Data from two small prospective studies suggest that MRI imaging can replace CT imaging during follow-up of patients with TC to reduce radiation exposure and diagnosis-induced secondary malignancy. Results from an ongoing randomized and one open-label study are expected soon and will aid in the decision-making of follow-up care of these patient.
